# Obesity-Mediated Regulation of HGF/c-Met Is Associated with Reduced Basal-Like Breast Cancer Latency in Parous Mice

**DOI:** 10.1371/journal.pone.0111394

**Published:** 2014-10-29

**Authors:** Sneha Sundaram, Alex J. Freemerman, Joseph A. Galanko, Kirk K. McNaughton, Katharine M. Bendt, David B. Darr, Melissa A. Troester, Liza Makowski

**Affiliations:** 1 UNC Nutrition Obesity Research Center, Gillings School of Global Public Health and School of Medicine, UNC Chapel Hill, Chapel Hill, North Carolina, United States of America; 2 Mouse Phase I Unit, Gillings School of Global Public Health and School of Medicine, UNC Chapel Hill, Chapel Hill, North Carolina, United States of America; 3 Lineberger Comprehensive Cancer Center, Gillings School of Global Public Health and School of Medicine, UNC Chapel Hill, Chapel Hill, North Carolina, United States of America; 4 Department of Cell and Molecular Physiology, Gillings School of Global Public Health and School of Medicine, UNC Chapel Hill, Chapel Hill, North Carolina, United States of America; 5 Departments of Epidemiology, and Pathology and Laboratory Medicine, Gillings School of Global Public Health and School of Medicine, UNC Chapel Hill, Chapel Hill, North Carolina, United States of America; 6 Department of Medicine, Gillings School of Global Public Health and School of Medicine, UNC Chapel Hill, Chapel Hill, North Carolina, United States of America; 7 Department of Nutrition, Gillings School of Global Public Health and School of Medicine, UNC Chapel Hill, Chapel Hill, North Carolina, United States of America; University of Alabama at Birmingham, United States of America

## Abstract

It is widely thought that pregnancy reduces breast cancer risk, but this lacks consideration of breast cancer subtypes. While a full term pregnancy reduces risk for estrogen receptor positive (ER+) and luminal breast cancers, parity is associated with increased risk of basal-like breast cancer (BBC) subtype. Basal-like subtypes represent less than 10% of breast cancers and are highly aggressive, affecting primarily young, African American women. Our previous work demonstrated that high fat diet-induced obesity in nulliparous mice significantly blunted latency in C3(1)-T_Ag_ mice, a model of BBC, potentially through the hepatocyte growth factor (HGF)/c-Met oncogenic pathway. Experimental studies have examined parity and obesity individually, but to date, the joint effects of parity and obesity have not been studied. We investigated the role of obesity in parous mice on BBC. Parity alone dramatically blunted tumor latency compared to nulliparous controls with no effects on tumor number or growth, while obesity had only a minor role in further reducing latency. Obesity-associated metabolic mediators and hormones such as insulin, estrogen, and progesterone were not significantly regulated by obesity. Plasma IL-6 was also significantly elevated by obesity in parous mice. We have previously reported a potential role for stromal-derived hepatocyte growth factor (HGF) via its cognate receptor c-Met in the etiology of obesity-induced BBC tumor onset and in both human and murine primary coculture models of BBC-aggressiveness. Obesity-associated c-Met concentrations were 2.5-fold greater in normal mammary glands of parous mice. Taken together, our studies demonstrate that, parity in C3(1)-T_Ag_ mice dramatically reduced BBC latency compared to nulliparous mice. In parous mice, c-Met is regulated by obesity in unaffected mammary gland and is associated with tumor onset. C3(1)-T_Ag_ mice recapitulate epidemiologic findings such that parity drives increased BBC risk and potential microenvironmental alterations in c-Met signaling may play a role in etiology.

## Introduction

Epidemiologic and experimental data have shown that a full term pregnancy reduces breast cancer risk [1]. However, with the advent of protein and mRNA expression profiling and the Tumor Cancer Genome Atlas (TCGA), classification of tumor subtypes with specific risks and outcomes has shed new light on breast cancer incidence [2], [3]. BBC represents 5–10% of breast cancers [4]. BBCs are estrogen receptor-, progesterone receptor- and human epidermal growth factor receptor 2 (HER2)- negative, thus, often referred to as triple-negative breast cancers, and as such these cancers lack a targeted therapy [5]. Patients have poor overall survival because these tumors are highly proliferative. Tumors are diagnosed predominantly in young African-American women, particularly obese women [6]–[8]. While a full term pregnancy reduces risk for estrogen receptor-positive breast cancers, like the luminal subtype [1], parity is actually associated with *increased* probability of developing the more aggressive basal-like breast cancer (BBC) subtype [7], [9]–[12].

The choice of mouse model is critical in modeling breast cancer subtypes. Protective effects of pregnancy have been described in luminal subtype models wherein parity antagonized the effects of carcinogens such as dimethylbenzanthracene (DMBA) [13], [14] and ionizing radiation [15]. The protective effect of pregnancy can be mimicked by exogenous administration of high levels of estrogen and progesterone [16]. However, to date there have been no such studies on the effects of parity on BBC onset in murine models. We have shown that BBC is characterized by unique epithelial-stroma interactions, which likely play a role in etiology [17]–[22]. Gatenby and Gillies speculate that the origin of cancer may lie not in mutations within epithelial cells, but within acquired or somatic mutations in the mesenchymal cells that control tissue structure [23]. Thus, we hypothesized that pregnancy would induce long term changes such as inflammatory and metabolic alterations in the breast microenvironment that promote BBC [19], [24].

Parity is often associated with excess weight gain and retention of weight after delivery, which is especially true for African American women, who gain more weight than recommended by the Institute of Medicine and retain more of that weight postpartum [25]. Obesity is a well-known risk factor for many cancers [26], with heterogeneous effects on breast cancer risk when subtypes and/or menopausal status are taken into account. For breast cancer overall, results for which are dominated by the most prevalent luminal subtype, postmenopausal obesity is a risk factor, while premenopausal obesity appears to have modest protective effects [27]–[29]. However for BBC, body mass index and/or waist hip ratio are significantly associated with risk, *regardless of age or menopause status* [10], [30]–[33]. Using nulliparous C3(1)-T_Ag_ mice, a unique genetically engineered murine model (GEMM) of BBC [4], [34], we reported that obesity reduced latency and induced tumor cell aggressiveness, which is the first work in preclinical models paralleling human epidemiologic BBC findings [35]. Furthermore, we reported that weight loss prior to tumor onset reduced tumor progression [21]. Hence, the role of obesity in BBC is established through epidemiologic and experimental findings; however, the underlying mechanisms of obesity-induced risk remain uncertain.

Since obesity is known to drive BBC onset and obesity is often linked to parity, interactions with parity were investigated to gain further mechanistic insight. Using C3(1)-T_Ag_ mice, we examined the effects of parity and obesity on tumor latency and progression. Parity alone dramatically blunted tumor latency when compared to previous findings in nulliparous mice [35]. In parous mice, obesity had only a minor role in reducing mean latency compared to lean parous mice. We have previously shown that stromal-derived hepatocyte growth factor (HGF/scatter factor) and its cognate receptor c-Met correlated with obesity-induced BBC tumor onset in nulliparous mice [22] and were reduced with weight loss [21]. HGF also mediated *in vitro* measures of BBC-aggressiveness in murine and human primary coculture models [36], [37]. Herein, we demonstrate that in parous mice, c-Met is elevated by obesity in the normal mammary gland which correlated with tumor onset. Systemic measures of cytokines and hormones were not significantly different except for obesity-induced increases in Il-6. Thus, parity in C3(1)-T_Ag_ mice reduced latency to the same extent as obesity in nulliparous mice.

## Materials and Methods

### Reagents

c-Met goat anti-mouse antibody (detects pro- and cleaved c-Met) was obtained from R&D Systems (Minneapolis, MN). Anti-SV40-T_Ag_ was obtained from Santa Cruz (Santa Cruz, CA). Estrogen and progesterone ELISA kits were obtained from Novatein Bio (Cambridge, MA).

### C3(1)-T_Ag_ Mouse Model

*Diets:* C3(1)-T_Ag_ mice [34] were used to study the role of obesity and parity on BBC. Studies were performed with approval and in accordance with guidelines of the Institutional Animal Care and Use Committee at the University of North Carolina at Chapel Hill (UNC-CH, NC). Female C3(1)-T_Ag_ mice were obtained through a collaboration with the UNC Lineberger Comprehensive Cancer Center (LCCC) Mouse Phase I Unit (MP1U). Female mice at seven weeks of age were placed with male mice for breeding. Males were removed at pregnancy. Following the delivery of litters, at maternal age of approximately 10 weeks old, pups were removed immediately after birth, and the mothers were randomly assigned to diet groups. Ten percent kcal from fat diet (“10%”) or 60% kcal (“60%”) lard-based diets matched for protein, vitamins, and minerals were obtained from Research Diets Inc. (New Brunswick, NJ) after customization, as in [36]. Custom diet information can be found in Table S1.

#### Tumor latency, number, growth and volume

Mice were monitored for tumor development by palpating thrice weekly following initiation of diets at 10 weeks of age. Initial tumor latency was defined as age at detection of first tumor and is reported as mean ± standard deviation (Table 1). Total tumor latency was defined as latency of all tumors palpated until sacrifice and is reported as Kaplan-Meier plot. Tumor volumes were measured once weekly over 3 weeks, following detection of first tumor, using ultrasound measurements with the Visualsonics 2000 (Toronto, Canada)) as in [36]. The tumor volumes were calculated using the formula: length×width^2^×0.5. The percent change in volume over time was calculated: (End volume – start volume)/Start volume * 100. The number of tumors per mouse was counted at sacrifice.

**Table 1:** Tumor latency in C3(1)-T_Ag_ nulliparous and parous mice.

Table 1	Nulliparous [35]	Parous	p-value
10%	18.99±0.59	16.50±0.38	0.001
60%	16.94±0.51	15.91±0.47	>0.05

Initial tumor latency was defined as the age at which first tumor palpated. Mean ± standard error of mean of tumor latency of the first tumor palpated. N = 15 per group.

#### Body weight and composition

Prior to starting mice on diet and weekly until sacrifice, body weight was measured in grams. Body composition including lean mass, fat mass, free water content and total water content of non-anesthetized mice was measured prior to initiating diet and monthly thereafter using the EchoMRI-100 quantitative magnetic resonance whole body composition analyzer (Echo Medical Systems, Houston, TX). Obesity is defined as greater than a 5% incremental increase in fat composition. Fat mass is presented as % fat mass over total body weight measured day of MRI.

#### Blood glucose

Blood glucose was measured prior to start of diet and at sacrifice following a 6 h fast using a Bayer Contour Blood Glucose Monitor (Bayer HealthCare LLC, Tarrytown, NY).

#### Tissue harvest

3 weeks after detection of the first tumor, mice were sacrificed by an intraperitoneal (i.p.) injection of avertin (Fisher Scientific, Pittsburgh, PA). Following euthanasia, blood was collected by cardiac puncture in a tube with 10 µl of 0.05 mM EDTA. Plasma was collected by centrifuging blood at 5000×*g* for 5 min. Mammary glands without palpable or visible tumors were collected as normal unaffected gland, although atypia of ductal epithelium could be present in C3(1)-T_Ag_ mice after 8 weeks of age [34]. Portions of the tissues were placed into a cassette and formalin fixed for immunohistochemical (IHC) analysis.

#### Plasma hormone panel

Plasma collected at sacrifice was used for measuring metabolically relevant hormones and inflammatory mediators (insulin, IL-6, MCP-1 and TNF-α) using the Milliplex MAP Mouse Metabolic Hormone Magnetic Bead Panel in the Luminex MAGPIX system (EMD Millipore, Billerica, MA). The homeostasis model assessment-insulin resistance (HOMA_IR_) was used to calculate the approximate insulin resistance using the formula (glucose (mg/dl at sacrifice)×insulin (at sacrifice)/405) as previously described [21], [36]. Estrogen and progesterone plasma concentrations were measured using ELISA assays following the manufacturer’s protocol (Novatein Bio; Cambridge, MA).

### IHC of c-Met and SV40 T_Ag_ in Normal Mammary Glands and Tumors

IHC for c-Met and SV40 T antigen (T_Ag_) was performed in normal mammary glands and tumors using methods as described [36], [38]. Stained slides were scanned into the Aperio Scanscope CS system (Aperio Technologies, Vista, CA) at a magnification of 40X. Sections were then analyzed quantitatively using the Aperio Imagescope software: membrane IHC algorithm for c-Met quantification and the positive pixel counts for diaminobenzidine (DAB) staining in the color deconvolution algorithm for T_Ag_ as in [36]. N = 6 random areas from sections (n = 2 per mouse) were quantified and averaged per tumor per animal (n = 5 mice per diet exposure group). Images (40X) shown are representative.

### Statistical Analysis

Raw data are available in Table S2. Data in all cases are expressed as mean ± standard error of the mean (S.E.M.). Comparison of mean was carried out using a one-way analysis of variance (ANOVA) analysis with the SPSS (version 20) software (IBM SPSS Statistic 20.0, Armonk, NY). The level of significance was set at P<0.05. Kaplan-Meier analyses were conducted using GraphPad Prism 5 software to estimate tumor latency. Log rank and chi-square tests were used to investigate differences among groups. P values<0.05 were considered statistically significant.

## Results

### Parity and obesity reduce C3(1)-T_Ag_ basal-like tumor latency

To determine if parity or parity and obesity altered tumor onset in C3(1)-T_Ag_ BBC mice, age-matched parous mice were fed control (10% kcal from fat) or obesogenic (60% kcal from fat) diets. Parity significantly decreased latency in lean mice fed 10% diet compared to previous reports in nulliparous mice fed identical diets (Table 1 [36]). In lean control mice fed 10% diet, parity decreased latency by almost 2.5 weeks (Table 1, P = 0.001). However, in obese mice fed 60% diet, the effect of parity on latency was not significantly different compared to obese nulliparous mice.

**Figure 1:**
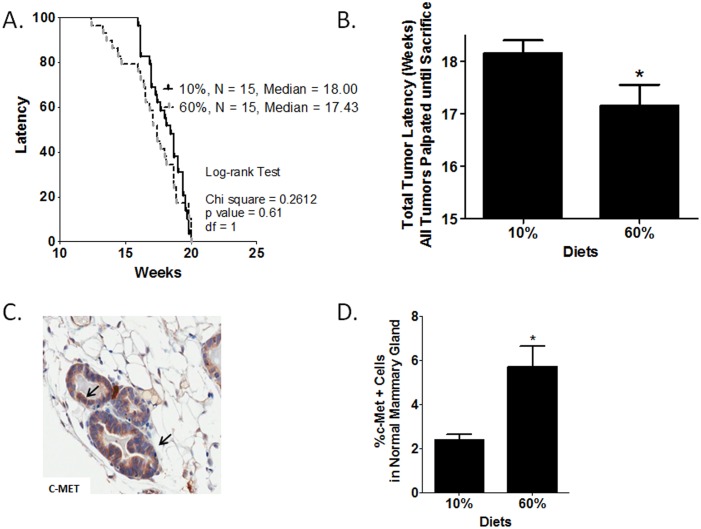
Obesity and parity regulated a decrease in BBC latency and elevated c-Met in normal mammary glands. A) Upon initiation of diet at 10 weeks of age, parous mice were palpated three times a week for tumor onset (first tumor detected) (N = 15). Using the Kaplan-Meier analysis the hazard ratio comparing 60% to 10% was 1.151 (95% CI of ratio: 0.67 to 1.98). Median latencies in 10% and 60%-fed mice were 18.00 and 17.43 weeks, respectively. Using a chi-square test with a degree of freedom of 1, 10% vs. 60% equaled 0.61. B) Mean latencies of total tumors palpated from initial until sacrifice are shown. N = 30 tumors in 15 mice per group. *P = 0.002. C) Representative 40X photomicrographs of the IHC analysis of membrane localized c-Met staining (arrows). D) Total c-Met protein levels in normal mammary glands were quantified. *P = 0.003.

Within parous mice, no significant effects were detected on median initial tumor latency (data not shown) or total tumor latency (initial (first tumor) plus subsequent tumors detected until sacrifice) (Figure 1A). The hazard ratio comparing median latencies of 18.00 weeks for 10%-fed mice to 17.43 for 60%-fed mice was 1.151 (95% CI of ratio: 0.67 to 1.98). However, obesity significantly reduced mean latency (Figure 1B, P = 0.002). There were no significant alterations in tumor number at sacrifice or tumor volume changes over three weeks from time of tumor identification until sacrifice in parous mice (data not shown).

### c-Met expression in normal mammary glands and tumors correlated with tumor latency

Significant effects of parity on latency but not tumor burden or progression in the C3(1)-T_Ag_ GEMM suggested that effects of parity were occurring early in tumorigenesis to significantly alter tumor onset. Previous work by our group has demonstrated a role for obesity-mediated HGF/c-Met signaling in normal mammary gland of nulliparous C3(1)-T_Ag_ mice [36] which was reversed by weight loss [21]. In the current study, normal mammary glands from parous mice were examined for c-Met receptor expression. c-Met expression in normal mammary gland from 60%-fed mice demonstrated primarily epithelial localization (Figure 1C). Digital quantification revealed that c-Met protein concentrations were significantly increased by 2.5-fold in obese 60% (P = 0.003)-fed mice compared with 10%-fed controls (Figure 1D). Interestingly in tumors, c-Met protein levels were significantly decreased in obese 60% (P = 0.042)-fed mice compared with 10%-fed controls (data not shown).

### Metabolic mediators, but not pregnancy hormones or T_Ag_ expression, in normal mammary glands and tumors associated with decreased latency

**Figure 2:**
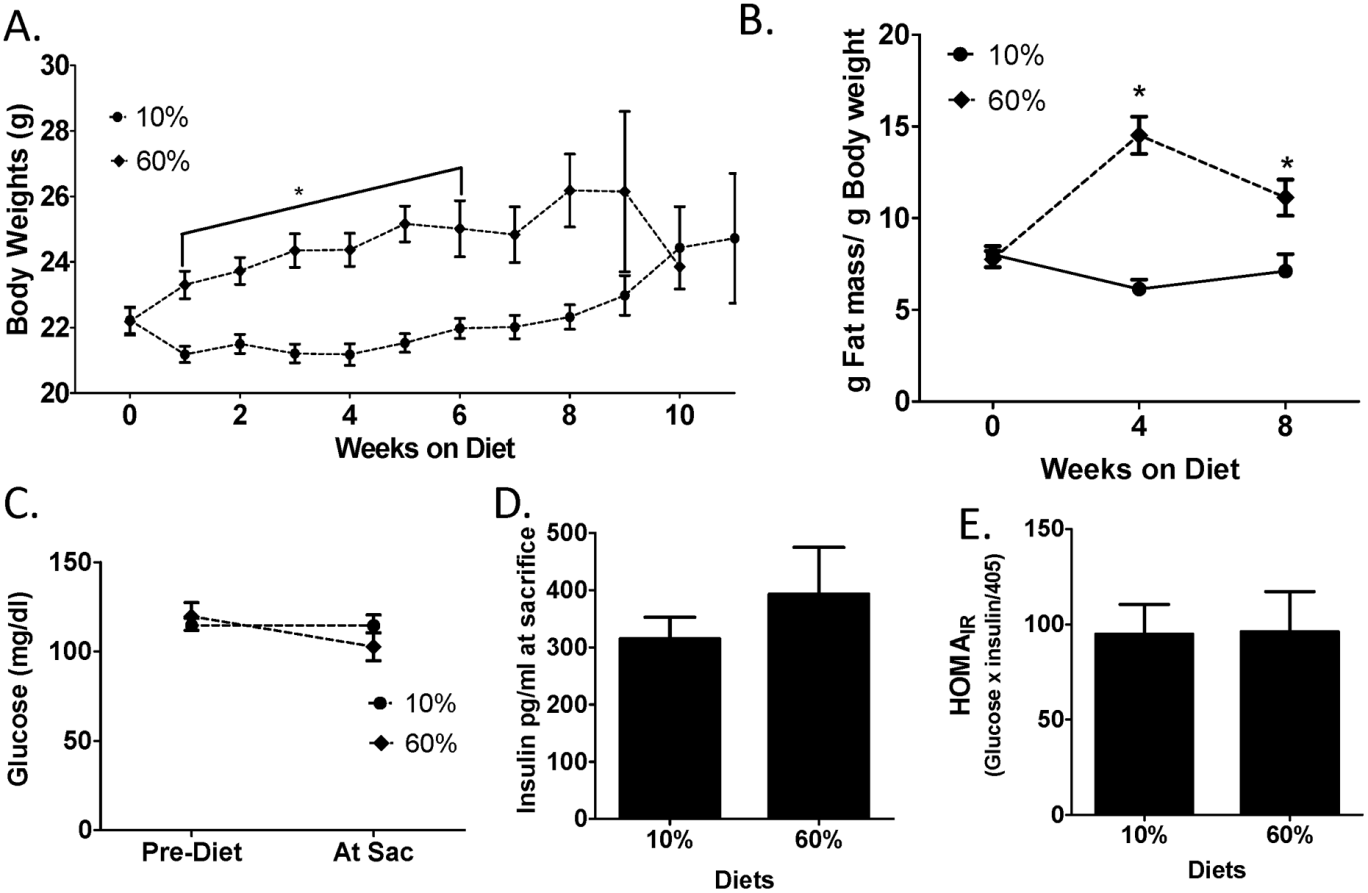
Diet-induced obesity affects body weight, adiposity, and leptin but not metabolic parameters in parous mice. A) C3(1)-T_Ag_ body weight was measured weekly over the course of the study until mice were sacrificed. Diet was initiated at 10 weeks of age (week 0 on diet) in n = 15 mice. *P<0.05 over weeks 1–6. B) Body fat content by MRI was measured monthly until sacrifice. Percent fat content over total body mass is shown. n = 15. *P<0.0001 at 4 weeks on diet, and P = 0.0049 at 8 weeks on diets. C) Blood glucose levels were measured from tail vein blood in mice fasted 6 hours. n = 15 mice. D) Insulin was measured at sacrifice in 6 hour fasted mice. n = 12. E) Homeostasis model assessment of insulin resistance (HOMA_IR_) was calculated from measures at sacrifice. n = 12.

To determine if metabolic parameters correlated with tumor onset, body weight and composition were measured. Parous mice fed 60% diets were significantly heavier compared with 10%-fed mice (P<0.05, Figure 2A). Compared to mice on 10% diet, 60%-fed mice also exhibited greater fat mass at 4 weeks on diet (P = 0.0001), and at 8 weeks (60% P = 0.0049, Figure 2B) as measured by MRI. Glucose, insulin and HOMA_IR_ measures did not vary by diet exposure in parous C3(1)-T_Ag_ mice (Figures 2C–E).

**Figure 3:**
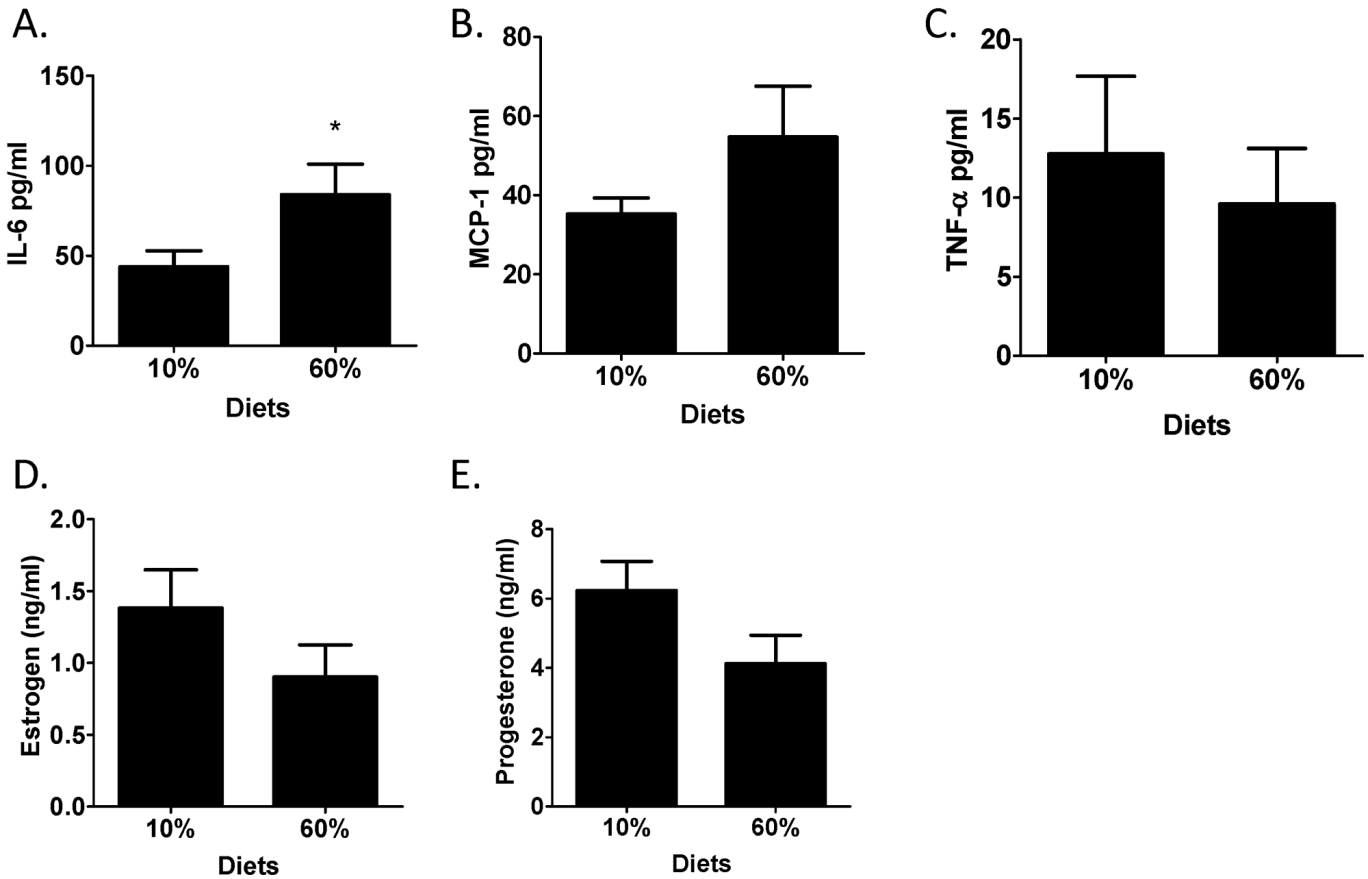
IL-6 concentration was elevated but plasma levels of other inflammatory mediators and reproductive hormones were not increased by obesity in parous mice. Cytokine and chemokine concentrations of A) IL-6, B) TNF-α and C) MCP-1 were measured in plasma at sacrifice. n = 12. *P = 0.028. D) Plasma estrogen levels were determined using ELISA analysis. n = 14. E) Plasma progesterone levels were determined using ELISA analysis. n = 14.

Systemic inflammatory mediators were also examined as potential contributors to tumor onset [35]. IL-6 was significantly elevated in obese mice (P = 0.03) compared to 10%-fed controls (Figure 3A). However, other chemokine and cytokines markers of systemic inflammation including MCP-1 and TNF-α were not modified by diet (Figure 3B and C). Estrogen and progesterone concentrations were similar between lean and obese mice (Figure 3D and E).

**Figure 4:**
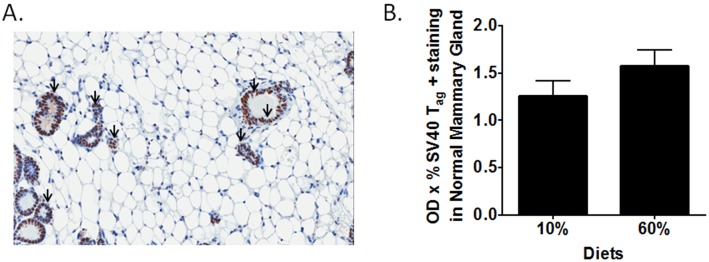
Normal mammary gland and tumor levels of SV40-T_Ag_ are not increased by obesity in parous mice. A) Representative 20X photomicrographs of SV40 T-antigen (T_Ag_) staining in normal mammary gland (A). Arrows indicate positive SV40 T_Ag_ staining. B) SV40 T_Ag_ levels in normal mammary glands (B) were quantified. n = 2 sections from 5 mice per diet group were digitally quantified. *P = 0.044.

To establish that parity or diet exposure did not alter expression of the oncogenic transgene SV40 T-antigen driven by the C3 promoter, which is the key driver of tumorigenesis in this model, IHC was undertaken and digitally quantified. No significant differences were observed in the SV40-T_Ag_ expression in the normal mammary glands (Figures 4A and B). In the tumors, there was in fact a significant decrease in the SV40 T_Ag_ expression levels in the 60% group compared with the 10% diet-fed mice (P = 0.044) (data not shown).

## Discussion

While pregnancy generally protects against the development of hormone-responsive estrogen and progesterone receptor positive tumors [39], it is an established risk factor for BBC. The Carolina Breast Cancer Study, among others, has identified parity as a positively associated risk factor for the development of BBC in both pre- and postmenopausal women [8], [9], [26], [34], [40], [41]. Similarly, while pre-menopausal obesity is associated with protection from the development of luminal breast cancers (the most prevalent subtype), both pre- and post-menopausal obesity are associated with increased BBC risk [7]. The convergence of pregnancy and obesity is an important public health concern in relation to BBC risk. During the reproductive years (approximately age 20–39), roughly 25% of non-Hispanic whites are obese (BMI≥30), and prevalence of obesity is approximately twice that in non-Hispanic black Americans (53.9% obese in 2003–2004) [42]. Since, obesity is a national epidemic in the United States [6] and BBC is a triple negative subtype that has no targeted therapies [5], it is important to understand the interaction between obesity and parity in relation to BBC risk.

Pregnancies cause extensive tissue remodeling, with the epithelium filling the majority of the mammary fat pad. Stromal cells including adipocytes, fibroblasts, and immune cells are important components of the mammary gland that work in concert to regulate the gland as it changes to produce milk during lactation. After weaning, tissue remodeling called involution causes the mammary gland to return to a microenvironmental architecture similar to the virgin gland [43], [44]. These developmental changes and alterations in microenvironment may represent opportunities for carcinogenesis and may further interact with other environmental exposures, such as dietary fat exposure and the obese state.

Using C3(1)-T_Ag_ mice, a BBC GEMM that represents human BBC [4], our previous work examining nulliparous mice demonstrated that obesity lead to a significant two week reduction in latency compared to lean controls [36]. Herein, we demonstrated that in lean mice parity alone significantly shortened BBC latency compared to nulliparous controls. However, in obese mice, the effect of parity was lost because parity did not reduce latency further than obesity alone. Within parous mice, obesity did not reduce median latency of the first tumor detected. However obesity significantly reduced secondary and subsequent mean tumor latency compared to lean controls. There were no effects on total tumor burden or promotion which was similar to findings in nulliparous mice [36]. Taken together, parity-induced reductions in latency may leave little opportunity for dramatic effects of obesity-associated factors that were observed in nulliparous mice. The C3(1)-Tag parous mouse model experiences tumorigenesis at a young age (akin to BBC in humans) [8], and thus poses a challenge for studying the joint effects of parity and obesity.

Obesity leads to numerous changes in the stroma of the mammary microenvironment and other fat pads including the release of growth factors and regulation of growth factor receptors [36], [38], [45]–[48]. The HGF/c-Met axis is a pathway that is linked to both obesity and breast cancer risk [21]. c-Met activation drives cell proliferation, angiogenesis, differentiation, migration, and anti-apoptosis pathways [49]–[51]. In nulliparous C3(1)-T_Ag_ mice, c-Met protein levels were significantly elevated by obesity in normal mammary gland and tumors [36]. Herein, we demonstrated that obesity also significantly elevated c-Met protein levels in the normal mammary glands in parous mice. However, cMet was not elevated by obesity in tumors with parity, although this was just a single measure on tumors isolated at sacrifice. It may be possible that obesity creates a dysfunctional microenvironment in the post-partum period wherein elevated c-Met protein levels persist in the normal mammary gland and are not down-regulated after resolution of involution, therefore creating fertile grounds for the development of HGF/c-Met-driven BBC. The role of c-Met signaling in BBC is currently under investigation. Future studies will be aimed at examining multiple time points from before tumor formation throughout tumor growth.

Insulin resistance has been hypothesized to increase breast cancer risk [52], [53]. In our study, obesity did not induce significant differences in hyperglycemia, hyperinsulinemia or insulin resistance indicating that these metabolic parameters do not play a role in the development of tumors in parous C3(1)-T_Ag_ GEMM. Likewise, there were no significant differences between nulliparous and parous mice, indicating that glucose and insulin plasma concentrations did not affect parity-induced reductions in latency. In this study, a significant elevation in systemic IL-6 concentrations in obese mice suggested a potential role for inflammation in tumor onset in parous C3(1)-T_Ag_ mice. Obesity did not induce significant differences in TNF-α and MCP-1 concentrations in parous mice. However, compared to the obese nulliparous mice [35], TNF-α concentrations were 5-fold greater in obese parous mice. Elevations in TNF-α and IL-6 in these mice suggest that inflammation is one pathway that may mediate effects of parity on reduced latency. Future studies examining local and systemic inflammation-mediated effects on BBC are needed to clarify the contribution to tumor onset. Finally, estrogen and progesterone concentrations likely do not contribute to the observed tumor latency differences induced by parity compared to nulliparous mice or obesity in parous mice.

In summary, our studies demonstrate that, similar to epidemiologic reports [7], parity dramatically reduced BBC latency compared to nulliparous mice and, importantly, obesity further accelerated subsequent tumor appearance. Obesity-elevated c-Met protein expression in normal mammary gland implicates a role for this growth factor pathway in the etiology of BBC. Further studies will test inhibition of c-Met signaling in the onset of post-partum obesity-driven BBC. Because BBC has unique risk factors that in many cases are traditionally thought of as breast cancer preventive factors [35], future studies should investigate the role of weight gain or loss in the post-partum period as modifiable BBC risk factors.

## Supporting Information

Table S1 **Contains information on custom diet.**
(DOCX)

Table S2 **Contains raw data.**
(XLSX)
