# Supplementary material for: Obesity-Mediated Regulation of HGF/c-Met Is Associated with Reduced Basal-Like Breast Cancer Latency in Parous Mice
Source: PLoS One. 2014 Oct 29;9(10):e111394. doi: 10.1371/journal.pone.0111394 (PMC4213021; doi:10.1371/journal.pone.0111394)
Supplement: Table S1 — Contains information on custom diet. (DOCX) [file pone.0111394.s001.docx]

| **Custom Product #** | **D11012202** | | **D11012204** | |
| --- | --- | --- | --- | --- |
| % | gm | *kcal* | gm | *kcal* |
| Protein | 19 | *20* | 26 | *20* |
| Carbohydrate | 67 | *70* | 26 | *20* |
| Fat | 4 | ***10*** | 35 | ***60*** |
| Total |  | *100* |  | *100* |
| kcal/gm | 3.8 |  | 5.2 |  |
|  |  |  |  |  |
| **Ingredient** | **gm** | ***kcal*** | **gm** | ***kcal*** |
| Casein | 200 | *800* | 200 | *800* |
| L-Cystine | 3 | *12* | 3 | *12* |
|  |  |  |  |  |
| Corn Starch | 575 | *2300* | 68.8 | *275* |
| Maltodextrin 10 | 125 | *500* | 125 | *500* |
|  |  |  |  |  |
| Cellulose, BW200 | 50 | *0* | 50 | *0* |
|  |  |  |  |  |
| Corn | 25 | *225* | 25 | *225* |
| Lard | 20 | *180* | 245 | *2205* |
|  |  |  |  |  |
| Mineral Mix S10026 | 10 | *0* | 10 | *0* |
| DiCalcium Phosphate | 13 | *0* | 13 | *0* |
| Calcium Carbonate | 5.5 | *0* | 5.5 | *0* |
| Potassium Citrate, 1 H20 | 16.5 | *0* | 16.5 | *0* |
|  |  |  |  |  |
| Vitamin Mix V10001 | 10 | *40* | 10 | *40* |
| Choline Bitartrate | 2 | *0* | 2 | *0* |
|  |  |  |  |  |
| FD&C Yellow Dye #5 | 0.05 | *0* | 0 | *0* |
| FD&C Red Dye #40 | 0 | *0* | 0 | *0* |
| FD&C Blue Dye #1 | 0 | *0* | 0.05 | *0* |
|  |  |  |  |  |
| **Total** | **1055.05** | ***4057*** | **773.85** | ***4057*** |

**Supplemental Table 1:** Custom purified diet components (Research Diets Inc.)
